# Effects of a Phone Call Intervention to Promote Adherence to Antiretroviral Therapy and Quality of Life of HIV/AIDS Patients in Baoshan, China: A Randomized Controlled Trial

**DOI:** 10.1155/2013/580974

**Published:** 2013-01-17

**Authors:** Dongsheng Huang, Rassamee Sangthong, Edward McNeil, Virasakdi Chongsuvivatwong, Weibin Zheng, Xuemei Yang

**Affiliations:** ^1^Department of Preventing and Controlling HIV/AIDS, Baoshan Prefecture Center for Disease Control and Prevention, Baoshan, Yunnan 678000, China; ^2^Epidemiology Unit, Faculty of Medicine, Prince of Songkla University, Hat Yai 90110, Thailand; ^3^Baoshan Prefecture AIDS Working Committee Office, Baoshan Prefecture Health Bureau, Baoshan, Yunnan 678000, China

## Abstract

*Background*. Suboptimal adherence to antiretroviral therapy (ART) is still pervasive. The effect of using a mobile phone call intervention to improve patient adherence is currently not known. *Objective*. This study aims to investigate the effects of a phone call intervention on adherence to ART and quality of life (QOL) of treatment-naive and treatment-experienced patients. *Methods*. A randomized controlled trial was conducted in the three largest public hospitals. Adherence was measured by self-completed questionnaires. QOL was assessed by the WHOQOL-HIV BREF. Outcomes were assessed at day 15, at 1, 2, and 3 months after start of treatment for treatment-naive patients and at 3 months after study enrollment for treatment-experienced patients. *Results*. A total of 103 treatment-naive and 93 treatment-experienced HIV/AIDS patients were consecutively recruited. Results show that a phone call intervention could maintain high self-reported adherence among both treatment-naive and treatment-experienced patients. After three months, significant QOL improvements were observed in domains of physical health (*P* = 0.003), level of independence (*P* = 0.018), environment (*P* = 0.002), and spirituality/religion/personal beliefs (*P* = 0.021) among treatment-naive patients. *Conclusion*. A mobile phone call intervention to patients could maintain high adherence rates although no statistically significant differences were found. A phone call could improve some domains of QOL among treatment-naive patients.

## 1. Introduction 

Since the beginning of the HIV/AIDS epidemic in 1981, nearly 30 million people have died from AIDS-related causes [1]. Antiretroviral therapy (ART) was initiated in 1996 and has contributed greatly to the reduction in mortality and morbidity among patients with HIV/AIDS [2]. The annual number of people dying from AIDS-related causes worldwide has been steadily decreasing from a peak of 2.2 million in 2005 to an estimated 1.8 million in 2010 [3]. In China, the mortality rate has decreased from 39.3 per 100 person-years in 2002 to 14.2 per 100 person-years in 2009 [4]. 

While the benefits of ART are well established, poor adherence to the regimens is pervasive [2]. Nonadherence rates range from 30% to 40% in the United States of America, Latin America, and Europe [5–8], and in China, it is approximately 20% [9]. An adherence rate under 95% accounts for ART failure and results in drug resistance [10]. Thus, it is crucial for patients with HIV/AIDS to consistently comply with the regimen to decrease the risk of disease progression and drug resistance, maintaining CD4 lymphocyte count to prolong their survival and to achieve a better quality of life (QOL) [11, 12].

Mobile phone technology using text messages has been shown to be useful to improve adherence rates [13, 14]. However, patients sometimes do not receive the messages, or if they do, ignore them [15]. Previous studies reported that participants prefer interventions that not only act as a reminder but also as a useful source of information on HIV/AIDS as well as allowing them to enquire about their illness or simply to communicate with their providers [13, 16]. A phone call intervention [17] has been shown to have promising results in developed countries and has been suggested to be used to improve adherence to ART in resource-limited countries. 

China is currently the world's largest mobile phone market [18], and approximately 76% of the population now own a mobile phone [19]. The present study was therefore carried out to investigate the effect of a phone call intervention to promote adherence to ART and QOL among HIV/AIDS patients in Baoshan, China.

## 2. Materials and Methods

### 2.1. Study Setting

Baoshan is located in the south western part of Yunnan, close to the Myanmar border, where HIV/AIDS is highly prevalent [20, 21]. Baoshan prefecture began to monitor the HIV/AIDS epidemic in 1990. At present, the accumulative number of living cases is totally more than 4,500, and more than 1,500 patients have been under ART (Baoshan CDC internal statistics). This randomized controlled trial (RCT) was conducted in 3 out of 6 county hospitals in Baoshan Prefecture in 2011.

### 2.2. Study Sample

Based on the national guideline for ART in China [22], patients are indicated for ART when they have (1) a positive confirmatory test for HIV; (2) baseline CD4 count <350 cells/mm^3^ or baseline WHO clinical staging of III or IV; (3) detailed history and physical examination. These patients were eligible to our study if they were (a) Chinese, (b) at least 18 years of age, (c) owning a mobile phone, (d) not imminently transferring to other hospitals, (e) not a prisoner, pregnant woman, or a hospitalized patient. Recruited patients were classified into two groups, treatment-naive patients and treatment-experienced patients. The latter group have been on treatment for 1 to 3 years. The required sample size was estimated to be at least 75 patients per study arm to detect an improvement of 20% in adherence rate from 75% to 95%, with 80% power, 95% confidence, and allowing for a 20% loss to followup. 

Eligible patients were consecutively asked to participate in the study. Details of the study were given to patients who agreed to participate in the study, and verbal informed consent was obtained. Treatment-naive and treatment-experienced patients were allocated to either the intervention or control groups by the permuted block of two and four randomization method. The study arm assignment was concealed in an opaque-sealed envelope. If the phone call intervention was allocated, the patient then received information regarding the intervention for 3–5 minutes by a well-trained registered nurse or health personnel.

### 2.3. Procedures

Two registered nurses or health personnel in each county hospital who had worked at the HIV/AIDS clinic were trained for 2 hours on usual care service including provision of ART information and instructions for taking medicine, general health care and self-management of symptoms related to HIV/AIDS and treatment, and relevant social support. Moreover, the nurse or health personnel who was designated to give the phone call intervention received counseling on stress-related problems, exploring patients' demands and expectations, and how to conduct a telephone appointment and interview, particularly regarding the privacy of the phone call and confidentiality of information. 

On the first visit, patients were assessed on their baseline characteristics, attitudes, knowledge, and readiness for treatment by an interview. Relevant clinical information including CD4 count was obtained from the patient's medical record. QOL was examined by the WHOQOL-HIV BREF [23] to obtain six domains of QOL, namely, physical well-being, psychological status, level of independence, social relationships, environment and spirituality/religion/personal beliefs.

### 2.4. Intervention

All patients received usual care services including education on HIV/AIDS and treatment. Questions and health problems could be discussed with the doctor at the clinic. In addition to the usual care, patients in the intervention group were given a hospital phone number and a mobile phone number. The patient's mobile phone number(s) was also recorded. A test call was made, and the date and time for the telephone calls were designated to each patient. A reminder phone call was planned to be made every two weeks except for when a patient arrived for their scheduled follow-up visit. At the start of the call, the patient's name would be identified before any conversation begun. The conversation would be made in a friendly and private manner. A semistructured dialogue eliciting the reasons and difficulties in making a hospital visit, symptoms of treatment, treatment adherence, and difficulty in taking medication was used. Any questions and concerns about medication, health, and related issues were welcome for patients to ask. Each phone would take about 3 minutes. All phone calls and relevant conversation would be noted. If a patient did not answer the phone or another person answered the phone, or it was not convenient for a patient to talk, up to 4 calls on the same day, the call would be attempted later. A successful call referred to a patient was reached and conversation was made.

### 2.5. Followup

Based on the national guidelines for HIV/AIDS treatment [22], HIV/AIDS patients were appointed for followup at 15 days, at 1, 2, and 3 months, and then every three months after start of treatment. In this study, patients were followed up for 3 months as designated in the routine followup. Treatment adherence, symptoms related to HIV/AIDS, reasons for not taking medication, if any, QOL, and clinical information were obtained at every followup visit. CD4 count was obtained at baseline and at the 3-month follow-up visit only. Clinical information including treatment details such as WHO clinical stage and weight was obtained from the medical records.

### 2.6. Ethical Consideration

All participants were given details of the study, and verbal informed consent was obtained. Privacy and confidentiality were ensured. Data were kept in a locked drawer and were accessible for only responsible registered nurses or health personnel. The study was approved by the Ethics Committee, Faculty of Medicine, Prince of Songkla University, Thailand and Baoshan Prefecture Health Bureau, China. The study was registered to http://clinicaltrials.gov/, NCT01395771.

### 2.7. Statistical Analysis

Descriptive statistics were used to compare baseline characteristics between intervention and control groups. Chi-square test or Fisher's exact test and Student's *t*-test were used to compare categorical variables and continuous variables between the two groups. Adherence rate in this study was computed by the percent of the prescribed drug 1 *‒* proportion pills missed × 100% [17]. McNemar's test was used to assess the change in proportion of WHO clinical staging of HIV/AIDS from pre- to postintervention. All data analysis was based on intention-to-treat. Data entry was entered in EpiData version 3.1 [24] and analysed by R version 2.14.0 [25]. 

## 3. Results

### 3.1. Flow of Participants

**Figure 1:**
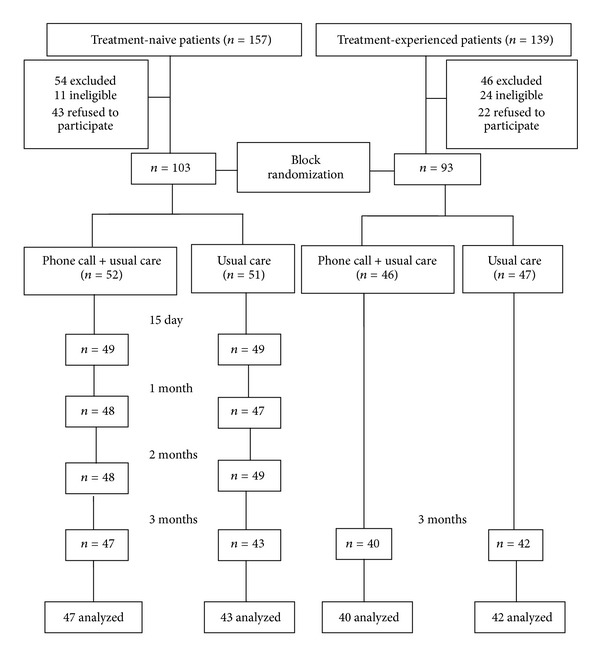
Flow chart of study participants.

Figure 1 shows that among the 157 treatment-naive patients, 54 were excluded (11 ineligible, 43 refused to participate). Thus, 103 treatment-naive patients were recruited. Among 139 treatment-experienced patients, 46 were excluded (24 ineligible, 22 refused). Thus, 93 patients were recruited. The primary reasons for refusal to participate in the study were not having time and having transportation difficulties. After randomization, 52 and 51 treatment-naive, and 46 and 47 treatment-experienced patients were allocated to the intervention and control groups, respectively. The overall follow-up rates at day 15 and months 1, 2, and 3 for treatment-naive patients were 95.1%, 92.2%, 94.2%, and 87.3%, respectively, while the follow-up rate at 3 months among treatment-experienced patients was 88.2%. 

### 3.2. Description of Participants

Table 1 shows the comparison of baseline characteristics of participants between the intervention and control groups. Males and females were equally distributed among the two groups and most were aged between 20 and 40 years. Most were Han ethnicity, married or cohabiting, had at least a primary school education, were farmers, and resided in rural areas. The average annual income was below 10,000 Chinese yuan (CNY) (1,577 USD); however, one-third of treatment-naive patients in the control group had an annual income above 10,000 CNY. 

Approximately 90% of all participants had a history of heterosexual contact, while only a few had a history of injecting drug use as a possible route of HIV infection. The average baseline CD4 count ranged from 200 to 350 cells/mm^3^; however, approximately one-third of treatment-experienced patients had a CD4 count above 350 cells/mm^3^. Most patients were WHO clinical stage I, and most patients received the standard regimen, AZT + 3TC + NVP/EFV, for treatment.

### 3.3. Mobile Phone Calls

Table 2 shows details of the bi-weekly phone calls made over the 12-week study period. A total of 231 and 564 phone calls were made for patients in the treatment-naive and treatment-experienced groups, respectively. The overall success rate for treatment-naive patients was 81.7%. The success rate was above 60% among treatment-experienced patients and was consistent across all call schedules. The average duration of phone calls was 2.9 ± 1.9 minutes and 2.4 ± 2.0 minutes in the treatment-naive and treatment-experienced groups, respectively. Conversations conducted over the phone were mostly related to clinical symptoms that patients had experienced recently and side-effects of their treatment.

### 3.4. Follow-Up Rate, Treatment Adherence, and Clinical Outcomes

Table 3 compares the follow-up rates, self-reported adherence to treatment, CD4 count, and weight change between the two groups at each visit. The rates tended to be lower among treatment-naive patients, although no significant differences were observed. Among treatment-experienced patients, the 3-month follow-up rate was slightly higher in the intervention group, although, again, no significant difference was observed. The adherence rate among treatment-naive patients in the intervention group was consistently above 98%, whereas it fluctuated slightly in the control group. There were no statistically significant differences in adherence rate, mean CD4 count, weight change, WHO clinical staging, and opportunistic infections between the intervention and control groups in both treatment-naive and treatment-experienced patients.

### 3.5. Quality of Life

Table 4 compares the mean WHO-QOL scores in 6 domains and change from baseline to month 3 between the two groups. In all QOL domains, the scores gradually improved over 3 months in both treatment-naive and treatment-experienced patients. Among treatment-naive patients, the mean QOL scores at baseline among the intervention and the control groups were similar. However, the scores after 3 months were significantly higher among patients who received the phone call intervention in domains of physical well-being, level of independence, environment, and spiritual/religious/personal beliefs. No significant differences in change in QOL scores were observed among the treatment-experienced patients.

## 4. Discussion

Results of this RCT show that although a 3-minute mobile phone call intervention had no significant effect on the follow-up rate, adherence to treatment, and clinical outcomes, it could help maintain patient follow-up visits and treatment adherence. The adherence rates in the control group tended to decrease over time. After three months of intervention, significant improvements were observed in QOL domains for physical well-being, level of independence, environment, and spiritual/religion/personal beliefs among treatment-naive patients. 

Many interventions have been trialed to promote and maintain adherence rates among patients with HIV/AIDS. Counseling and educational programs have shown to offer no or only minimal improvements to adherence rates [26, 27], while directly observed therapy (DOT) seems to offer no benefit over self-administered treatment [28]. Moreover, DOT is labor intensive, expensive, and can be perceived as intrusive [29]. Monetary incentives have been shown to offer positive reinforcement, but adherence rates are not sustained and return to baseline levels after discontinuation of the intervention [30]. 

Subsequently, a variety of electronic devices, such as pagers [31], disease management assistance system (DMAS) [32], electronic pill-cap [33], and alarms, have been used to regularly remind patients to take their medication [34]. A recent systematic review [35] confirmed that these devices may result in improved adherence to ART. However, these devices have drawbacks, including inconvenience, high cost, and patient dissatisfaction due to privacy concerns [36, 37]. Moreover, the devices solely serve as a reminder and do not cope with potential adherence barriers, including management of side effects, provision of social support, and allowing the patient to discuss their health problems. 

A previous randomized trial in the USA [38] on a scripted serial landline telephone call among treatment-naive HIV/AIDS patients over 96 weeks showed higher self-reported adherence rates in the telephone group but no statistically significant improvements in virologic outcomes. A subsequent randomized trial in the USA [17] assessing the effect of an 8-minute landline telephone call among treatment-naive patients showed a significantly higher adherence rate at week 64 in the telephone group as well as being able to maintain high adherence rates over time. 

Recently, mobile technology for health (mHealth), such as mobile phone use, is a new promising innovative option in health care [39, 40]. It can surmount barriers such as stigma, loss of privacy, and transportation limitations associated with traditional interventions [13]. The feasibility of mobile phone use in health care among patients with HIV/AIDS has been demonstrated in both developed and resource-limited countries [13, 14, 41–43]. Mobile phone technology can offer medication reminders via a short message service, voice call, dissemination of information, and communication with health workers. However, some studies [15, 44] have reported that a reminder can easily be ignored. Patients prefer to obtain information and be given the opportunity to communicate with health workers. 

Our study is one of the first RCTs to assess mobile phone use [14, 41, 45] on adherence rates among patients with HIV/AIDS. A weekly mobile phone message over 12 months in Kenya [14] was found to improve patient adherence rates. Two other protocols [41, 45] are currently in progress, and the results will hopefully offer a better understanding of the use of this technology. In addition to reminding services as provided in other RCTs [14, 41, 45], our study offered a combination of reminder, information dissemination, and communication with health workers through a biweekly mobile phone call service. Although adherence rates were not significantly better in the intervention group, they were sustained across all follow-up visits among treatment-naive patients. The usefulness of mobile phone intervention may thus be confined to treatment-naive patients. Those patients lack knowledge and experience in ART and self-care and have not yet developed their own coping mechanisms, unlike treatment-experienced patients. The questions most commonly asked by patients related to symptoms experienced, such as poor appetite, fever, fatigue, side-effects and toxicity of the treatment, and problems with opportunistic infection. Similarly, a previous pilot study [44] on mobile phone surveys for HIV/AIDS care in southern India reported that most calls were related to ART toxicity, ART initiation, and management of opportunistic infections. 

In addition to adherence rate, QOL is an important concern in health care and treatment provision. While many electronic devices have been invented, primarily to improve adherence rates, they showed no improvement in QOL [34, 37]. For example, the DMAS [37] provides a verbal reminder of the medication time but results in deterioration of the patient's QOL which may be due to a violation of the patient's privacy by the reminder, explained by the authors. 

QOL has not yet been evaluated in other RCTs where mobile phone use was used as the intervention [41, 45], whereas the mobile phone call in our study supported a better QOL among treatment-naive patients. Similarly, a recent intervention study investigating home visits and a telephone intervention conducted in China [46] reported improvement of adherence rate and QOL for physical, psychological, social, and environmental domains. Stand-alone devices which provide reminders but lack the ability to allow patients to communicate with health personnel may improve adherence rates, but they cannot improve the QOL of patients with HIV/AIDS. Previous studies [13, 16] reported that patients prefer interventions which not only act as a reminder but also provide a source of useful information on HIV/AIDS as well as allowing them to communicate with healthcare providers. 

Although this study had a strong study design, the sample size was too small to detect differences in adherence rates between the intervention and the control groups. The post hoc power of the study ranged from only 40% to 60% across follow-up visits. High self-reported adherence rates in our study were observed in both the intervention and control groups. Although the high adherence to ART in China has also been reported in another study [47], this may also be a result of the Hawthorn effect where patients tend to modify their behaviors or response under close observation and assessment while participating in a study. Due to budget constraints, viral loads were too expensive to be monitored. A larger study with longer follow-up duration assessing adherence to treatment, QOL, and cost-effectiveness should be conducted to tailor the most suitable intervention in this setting.

## Figures and Tables

**Table 1:** Baseline characteristics of study participants in intervention and control groups.

Participant characteristic	Treatment-naive		Treatment-experienced	
	Intervention (*n* = 52)	Control (*n* = 51)	Intervention (*n* = 46)	Control (*n* = 47)
Sex (%)				
Male	24 (46.2)	27 (52.9)	22 (47.8)	21 (44.7)
Female	28 (53.8)	24 (47.1)	24 (52.2)	26 (55.3)
Age group (years)				
20–40	38 (73.1)	39 (76.5)	32 (69.6)	34 (72.3)
>40–60	14 (26.9)	10 (19.6)	13 (28.3)	13 (27.6)
>60	0 (0.0)	2 (3.9)	1 (2.2)	0 (0.0)
Ethnicity (%)				
Han	41 (78.8)	49 (96.1)	41 (89.1)	40 (85.1)
Other	11 (21.2)	2 (3.9)	5 (10.9)	7 (14.9)
Marital status (%)				
Single	3 (5.8)	2 (3.9)	3 (6.5)	2 (4.3)
Married/cohabiting	44 (84.6)	46 (90.2)	41 (89.1)	39 (83.0)
Divorced/widowed	5 (9.6)	3 (5.9)	2 (4.3)	6 (12.8)
Education level (%)				
None	4 (7.7)	3 (5.9)	3 (6.5)	3 (6.4)
Primary school	21 (40.4)	24 (47.1)	14 (30.4)	18 (38.3)
Junior high school	24 (46.2)	19 (37.3)	22 (47.8)	20 (42.6)
High school or above	3 (5.8)	5 (9.8)	7 (15.2)	6 (12.8)
Occupation (%)				
Agriculture	44 (84.6)	41 (80.4)	37 (80.4)	37 (78.7)
Business	6 (11.5)	7 (13.7)	3 (6.5)	2 (4.3)
Other	2 (3.8)	3 (5.9)	6 (13.0)	8 (17.0)
Residential area (%)				
Rural	47 (90.4)	48 (92.3)	39 (84.8)	43 (91.5)
Urban	5 (9.6)	4 (7.7)	7 (15.2)	4 (8.5)
Annual income (CNY)^a^				
<2000	22 (42.3)	18 (35.3)	14 (30.4)	9 (19.1)
2000–10000	21 (40.4)	15 (29.4)	24 (52.2)	31 (66)
>10000	9 (17.3)	18 (35.3)	8 (17.4)	7 (14.9)
Transmission route (%)				
Heterosexual	46 (88.5)	42 (82.4)	43 (93.5)	44 (93.6)
Injecting drug use	6 (11.5)	8 (15.7)	2 (4.3)	5 (10.6)
Other	4 (7.7)	8 (15.7)	1 (2.2)	1 (2.1)
CD4 count (cells/mm^3^)				
≤100	12 (23.1)	9 (17.6)	2 (4.4)	1 (2.2)
>100–200	13 (25.0)	10 (19.6)	10 (22.2)	8 (17.8)
>200–350	27 (51.9)	32 (62.7)	18 (40)	21 (46.7)
>350	—	—	15 (33.3)	15 (33.3)
WHO clinical staging (%)				
I	30 (57.7)	36 (70.6)	26 (56.5)	22 (46.8)
II	10 (19.2)	6 (11.8)	6 (13.0)	12 (25.5)
≥III or IV	12 (23.1)	9 (17.6)	14 (30.4)	13 (27.7)
ART regimen (%)				
AZT + 3TC + EFV^b^	19 (36.5)	15 (29.4)	9 (19.6)	11 (23.4)
AZT + 3TC + NVP^c^	28 (53.8)	31 (60.8)	26 (56.5)	23 (48.9)
Other	5 (9.6)	5 (9.8)	11 (23.9)	13 (27.7)

^a^1 USD = 6.36 CNY; ^b,c^AZT: Zidovudine; 3TC: Lamivudine; NVP: Nevirapine; EFV: Efavirenz; ART: antiretroviral therapy.

**Table 2:** Details of the biweekly phone calls made over the 12-week study period.

	Treatment-naive patients			Treatment-experienced patients		
Phone call appointment	No. of patients requiring a phone call *n* = 52	No. of successful calls *n* (%)	Call time (min) (mean ± SD)	No. of patients requiring a phone call *n* = 46	No. of successful calls *n* (%)	Call time (min) (mean ± SD)
Day 15	3	2 (66.0)	4.7 ± 1.2	46	32 (69.6)	2.5 ± 2.2
Month 1	4	3 (75.0)	6.0 ± 0.0	46	30 (65.2)	2.3 ± 2.2
Month 1.5	51	46 (90.2)	3.0 ± 1.8	46	28 (60.9)	2.2 ± 1.2
Month 2	6	2 (33.3)	1.0 ± 1.4	46	29 (63.4)	2.4 ± 2.1
Month 2.5	51	44 (86.2)	3.0 ± 1.8	46	29 (63.4)	3.1 ± 1.9
Month 3	5	1 (20.0)	3.0 ± 0.0	6	6 (100.0)	0.6 ± 1.3

Overall	120	98 (81.7)	2.9 ± 1.9	236	154 (65.3)	2.4 ± 2.0

**Table 3:** Follow-up rate, self-reported adherence to treatment, CD4, and weight change between intervention and control groups over time.

	Treatment-naive patients					Treatment-experienced patients				
	Group					Group				
					*P* value					*P* value
	*n*	Intervention	*n*	Control		*n*	Intervention	*n*	Control	
Follow-up rate (%)										

Visit 1	49	94.2	48	94.1	0.17	42	91.3	46	97.9	0.82
Visit 2	48	92.3	47	92.2	0.20		—	—		
Visit 3	48	92.3	49	94.2	0.10		—	—		
Visit 4	47	90.3	43	84.3	0.08	44	95.7	44	93.6	0.66

Self-reported adherence, mean (SD)										

Baseline	52	—	51	—		46	99.2 (2.3)	47	99.1 (2.5)	0.83
Day 15	40	98.9 (3.9)	36	97.3 (11.2)	0.98		—		—	
Month 1	29	98.7 (5.3)	19	94.7 (22.5)	0.19		—		—	
Month 2	32	99.8 (0.8)	33	97.0 (17.2)	0.98		—		—	
Month 3	30	99.7 (1.6)	31	96.5 (17.8)	0.09	40	99.6 (1.0)	42	99.5 (2.0)	0.37

CD4 (cell count/mm^3^) mean (SD)										

Baseline	49	191 (99.6)	47	216 (98.2)	0.23	39	286 (129.7)	40	348 (195.6)	0.10
Month 3	40	308 (124.3)	38	298 (121.9)	0.82	36	324 (125.0)	27	356 (197.5)	0.47
Pre- to postchange	40	111 (97.3)	38	91.9 (87.8)	0.35	36	32.1 (79.0)	27	12.3 (80.3)	0.34

Weight (kg) mean (SD)										

Baseline	49	54.1 (8.6)	47	56.7 (9.6)	0.15	46	57.3 (9.1)	47	53.4 (7.8)	0.02
Month 3	46	54.2 (9.3)	43	57.3 (9.4)	0.12	39	57.2 (9.4)	40	53.3 (8.0)	0.05
Pre- to postchange	46	0.9 (3.0)	43	0.6 (2.3)	0.61	39	0.2 (2.1)	40	0.04 (1.8)	0.77

**Table 4:** Comparison of quality of life domains and change (mean, SD) from baseline to month 3 between intervention and control groups.

	Treatment-naive patients					Treatment-experienced patients				
Domain					*P* value					*P* value
	*n*	Intervention	*n*	Control		*n*	Intervention	*n*	Control	
Physical well-being										

Baseline	52	12.6 ± 3.4	51	13.8 ± 2.6	0.044	46	14.3 ± 2.2	47	13.9 ± 2.8	0.478
Month 3	30	16.5 ± 4.0	31	15.8 ± 3.3	0.414	40	16.8 ± 2.5	42	16.3 ± 2.6	0.319
Change	30	4.3 ± 3.5	31	1.5 ± 3.7	0.003	40	2.55 ± 3.2	42	2.38 ± 3.4	0.817

Psychological status										

Baseline	52	11.7 ± 2.21	51	11.7 ± 2.2	0.875	46	12.1 ± 2.1	47	12.1 ± 2.0	0.886
Month 3	30	13.3 ± 2.14	31	12.9 ± 1.9	0.468	40	13.9 ± 1.7	42	13.0 ± 2.4	0.055
Change	30	1.6 ± 2.37	31	1.1 ± 2.8	0.485	40	1.78 ± 2.4	42	0.95 ± 2.5	0.126

Level of independence/work capacity										

Baseline	52	12.2 ± 2.8	51	13.2 ± 2.6	0.083	46	13.9 ± 2.7	47	13.1 ± 2.0	0.159
Month 3	30	13.3 ± 2.3	31	12.9 ± 1.5	0.465	40	13.7 ± 1.7	42	13.0 ± 1.7	0.054
Change	30	1.4 ± 2.5	31	−0.4 ± 3.2	0.018	40	−0.2 ± 2.4	42	−0.1 ± 2.1	0.949

Social relationships										

Baseline	52	13.2 ± 2.3	51	13.5 ± 2.4	0.477	46	13.3 ± 3.1	47	13.4 ± 2.4	0.864
Month 3	30	15.0 ± 2.2	31	14.4 ± 2.7	0.353	40	14.9 ± 2.2	42	14.3 ± 2.2	0.172
Change	30	1.8 ± 2.2	31	0.7 ± 3.3	0.114	40	1.7 ± 3.3	42	0.9 ± 2.2	0.214

Environment										

Baseline	52	11.7 ± 2.2	51	12.5 ± 2.4	0.072	46	12.0 ± 2.5	47	12.0 ± 2.7	0.983
Month 3	30	13.6 ± 2.2	31	12.7 ± 2.0	0.101	40	14.0 ± 2.3	42	13.1 ± 2.1	0.073
Change	30	1.8 ± 2.0	31	−0.03 ± 2.1	0.002	40	2.0 ± 2.6	42	1.1 ± 2.8	0.135

Spiritual/religious/personal beliefs										

Baseline	52	11.1 ± 3.5	51	12.4 ± 3.5	0.066	46	12.8 ± 3.7	47	12.0 ± 3.0	0.248
Month 3	30	15.5 ± 3.0	31	14.6 ± 3.6	0.297	40	16.2 ± 2.9	42	15.1 ± 3.3	0.123
Change	30	4.3 ± 2.9	31	2.4 ± 3.4	0.021	40	3.3 ± 4.5	42	3.1 ± 4.4	0.836
